
JOURNAL INFORMATION
==============================
NLM Title Abbreviation: Tex Med J (Austin)
NLM Journal ID: 21130590R

Texas Medical Journal

ISSN: 0892-8495
Publisher: Biomedical Journal Digitization Project

ARTICLE INFORMATION
==============================
PMCID: PMC9592027
Article version: 1
Subjects: Books and Magazines

Progressive Medicine, Vol. I., 1902

A Quarterly Digest of Advances, Discoveries and Improvements in the Medical and Surgical Sciences

Print publication date: 1902 Jul
Volume: 18
Issue: 1
First page: 37
Last page: 38
License: This work is free of known copyright restrictions. For more information, please see PMC Back Issue Digitization.
License URL: https://creativecommons.org/publicdomain/mark/1.0/

==============================

Phillips' Emulsion 50% best NORWAY COD LIVER OIL minutely sub-divided, WITH WHEAT PHOSPHATES (Phillips')

Pancreatized, Palatable, Permanent, Miscible in Water, Milk, Wine, etc.

Phillips' Milk of Magnesia Mg H2 O2 (FLUID.) "THE PERFECT ANTACID."

for correcting Hyperacid conditions—local or systemic.
Vehicle for Salicylates, Iodides, Balsams, etc.

Phillips' Phospho-Muriate of Quinine, COMP.

TONIC AND RECONSTRUCTIVE.

WHEAT PHOSPHATES, WITH MURIATE OF QUININE AND STRYCHNINE.

PHILLIPS' WHEAT PHOSPHATES (ACID).
PHILLIPS' SYRUP OF WHEAT PHOSPHATES.
PHILLIPS' DIGESTIBLE COCOA.

THE CHAS. H. PHILLIPS CHEMICAL CO., NEW YORK

authorities are given the prominence they deserve. The parts of the work dealing with materia medica and therapeutics, general medicine and surgery, X-rays, and sanitary science are all that could be desired. Every physician should be familiar with the facts set down in these sections. R.

---

Progressive Medicine, Vol. I., 1902. A Quarterly Digest of Advances, Discoveries and Improvements in the Medical and Surgical Sciences. Edited by Hobart Amory Hare, M. D., Professor of Therapeutics and Materia Medica in the Jefferson Medical College of Philadelphia. Octavo, handsomely bound in cloth, 452 pages, 5 illustrations. Per volume $2.50, by express prepaid to any address. Per annum, in four cloth-bound volumes, $10.00. Lea Brothers & Co., Philadelphia and New York.

In this number of Progressive Medicine Dr. Chas. A. Frazier reviews the advances made during the past year in the surgery of the head, neck and chest. He outlines the progress made in the surgery of the Gasserian ganglion and of the heart. Dr. Frederick A. Packard considers the infectious diseases, including acute rheumatism, croupous pneumonia and influenza. Dr. Floyd M. Crandall writes on Diseases of Children. He emphasizes the great importance of proper infant feeding. This section is excellent in every respect and will prove to be of great practical value as an aid to the general practitioner. The other sections, all of which are well written and exhaustive, are Pathology, by Dr. Ludwig Hectoen; Laryngology and Rhinology, by St. Clair Thompson, and Otology, by Robt. L. Randolph. R.

---
